# A Case of Hepatic Tuberculosis in a Patient on Adalimumab for Ankylosing Spondylitis

**DOI:** 10.7759/cureus.61264

**Published:** 2024-05-28

**Authors:** Shayan Amini, Ronan Allencherril, Michelle Lin, Suzanne M Crumley, David W Victor

**Affiliations:** 1 Internal Medicine, Houston Methodist Hospital, Houston, USA; 2 Gastroenterology and Hepatology, Houston Methodist Hospital, Houston, USA; 3 Pathology, Houston Methodist Hospital, Houston, USA; 4 Hepatology and Transplant Medicine, Houston Methodist Hospital, Houston, USA

**Keywords:** tumor necrosis factor-alpha (tnf-α) inhibitors, ankylosing spondilytis, adalimumab (humira), mycobacterium tuberculosis, extrapulmonary tuberculosis (eptb), transaminitis, hepatic tuberculosis (hepatic tb)

## Abstract

Hepatic tuberculosis (TB) is an uncommon extrapulmonary manifestation of tuberculosis. Hepatic TB is more common in immunocompromised patients, such as those on immunosuppressive medications or those with a human immunodeficiency virus (HIV) infection. Primary hepatic TB is rare, and liver involvement is often secondary to spreading from the lymphatics, portal vein, or hepatic artery. We report a case of hepatic TB in a patient on adalimumab for ankylosing spondylitis (AS).

## Introduction

*Mycobacterium tuberculosis* remains one of the most prevalent causes of morbidity and mortality in the world, affecting up to 10 million people per year [1]. Hepatic tuberculosis (TB) accounts for 1% of extrapulmonary cases of TB [1,2]. Hepatic TB is often asymptomatic or presents with nonspecific symptoms [2]. Rifampin, isoniazid, pyrazinamide, and ethambutol (RIPE) remain the treatment of choice for hepatic TB despite their potential hepatotoxicity [1]. Tumor necrosis factor-alpha (TNF-⍺) inhibitors are associated with a higher risk of infection with TB. As a result, all patients on anti-TNF-⍺ therapy should be monitored for symptoms of TB until six months after the completion of therapy [3]. We report a case of hepatic TB in a patient on adalimumab for ankylosing spondylitis (AS).

## Case presentation

**Figure 1:**
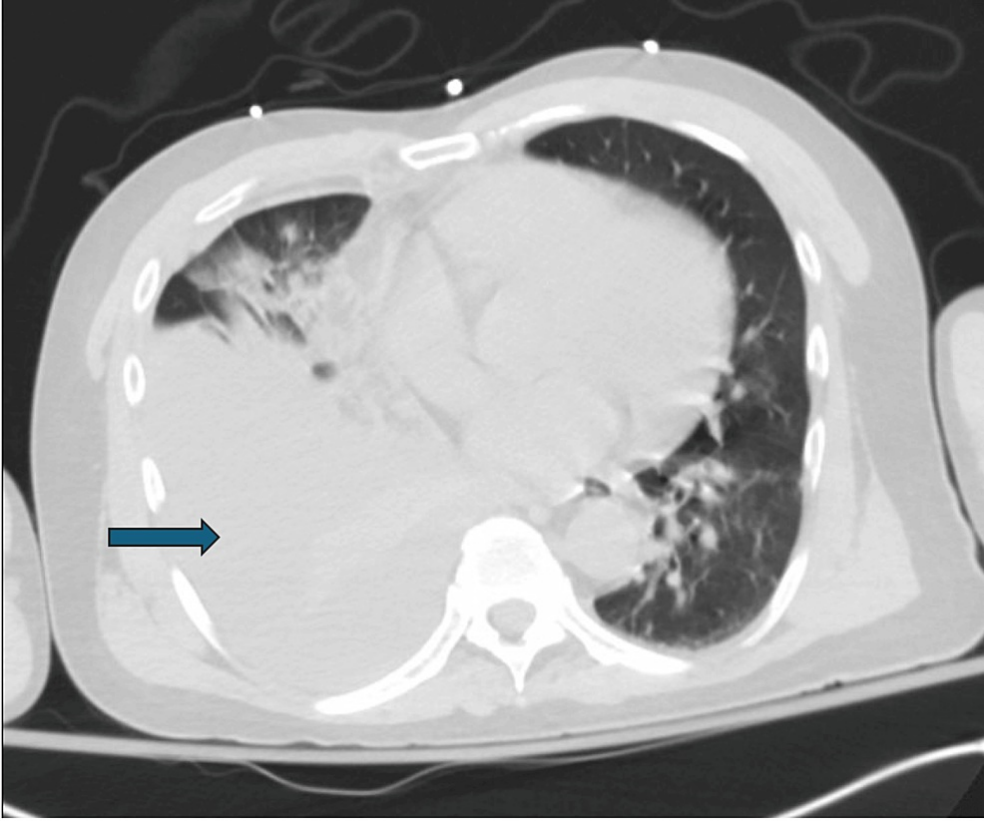
The initial CT scan of the chest reveals a moderately-sized right-sided pleural effusion.

A 52-year-old male with a medical history of AS on adalimumab and hypertension presented to the hospital with fever, cough, and shortness of breath. Two months prior to arrival, the patient had increased the dosing of his adalimumab to every week due to worsening arthralgia. Initially, the patient presented to an emergency department (ED) for similar symptoms and was treated for pneumonia with cefpodoxime and azithromycin. However, his symptoms worsened despite antibiotic therapy at home. Subsequent outpatient computed tomography (CT) of the chest ordered by the patient's primary care provider showed a right-sided pleural effusion (Figure 1).

**Figure 2:**
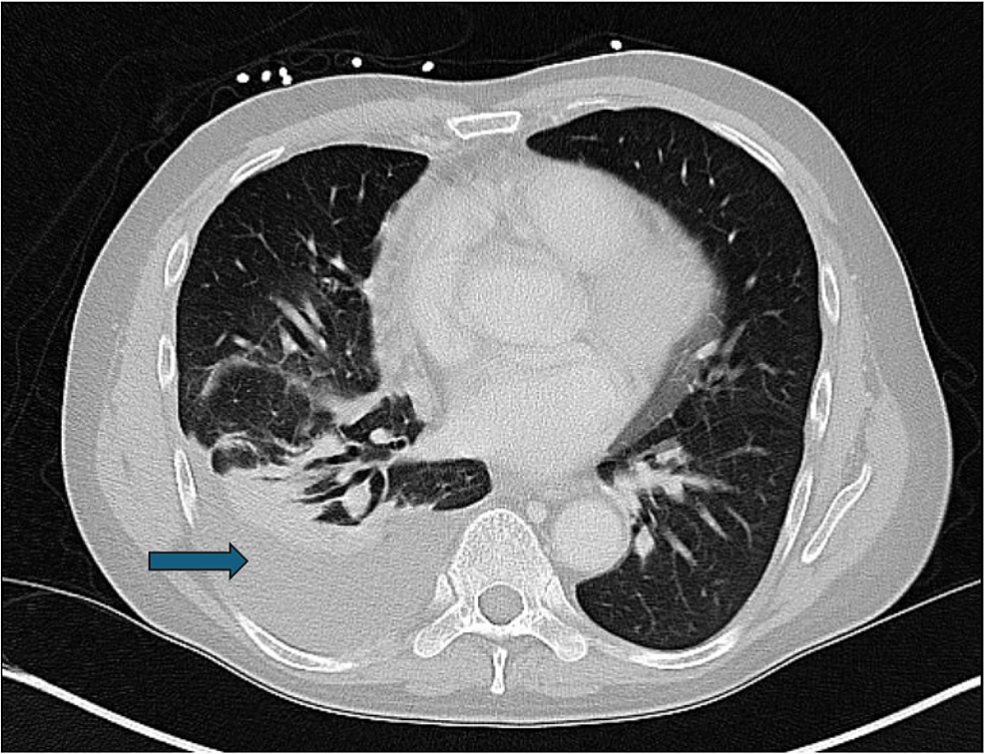
A repeat CT scan of the chest reveals the recurrence of a small-to-moderately-sized right-sided pleural effusion.

Laboratory results on presentation to the hospital revealed mild hepatitis with aspartate aminotransferase (AST) 162 U/L, alanine aminotransferase (ALT) 105 U/L, and alkaline phosphatase (ALP) 247 U/L. A chest X-ray confirmed the presence of a right-sided pleural effusion. The patient was empirically treated for sepsis secondary to pneumonia with vancomycin, cefepime, and doxycycline. Thoracentesis of the right-sided pleural effusion was consistent with an exudative effusion. The patient remained febrile despite antibiotic therapy. Bronchoscopy with bronchoalveolar lavage (BAL) was inconclusive, and the BAL cultures failed to yield any pathogens initially. The patient continued to remain febrile with shortness of breath. Throughout his course, the patient was noted to have persistent hepatitis with increasing ALT and AST. Lab testing did not reveal the clear cause of his hepatitis. The viral hepatitis workup was only positive for the hepatitis B surface antibody. The autoimmune hepatitis panel, including anti-smooth muscle antibodies, anti-mitochondrial antibodies, liver-kidney microsome antibodies, and immunoglobulins, was overall unremarkable. In addition, he was not on any medications that could explain his transaminitis. A hepatic ultrasound showed hepatomegaly without any mass or ascites. Due to concern for an associated autoimmune disease, a liver biopsy was considered but deferred given his concerning pulmonary status and lack of signs and symptoms of acute liver failure. He was started on intravenous corticosteroids with mild improvement in symptoms and hepatitis. However, his fever returned, and his liver function test (LFT) levels continued to rise. A repeat CT of the chest showed multifocal pneumonia with re-accumulation of the pleural effusion (Figure 2).

**Figure 3:**
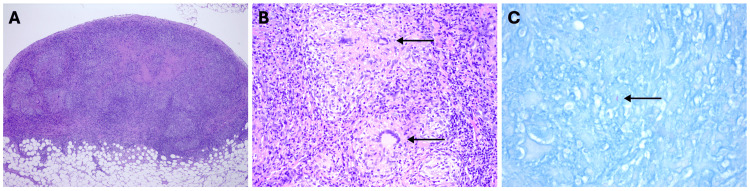
A pleural biopsy demonstrates extensive necrotizing granulomatous inflammation (image A) with scattered multinucleated giant cells (image B). Occasional acid-fast bacilli (AFB) are highlighted with the Kinyoun AFB stain (image C). (A: 40x magnification; B: 200x magnification; C: 600x magnification).

A pleural biopsy was performed, and pathology results were consistent with extensive necrotizing granulomatous inflammation with a positive acid-fast bacilli stain (Figure 3).

**Figure 4:**
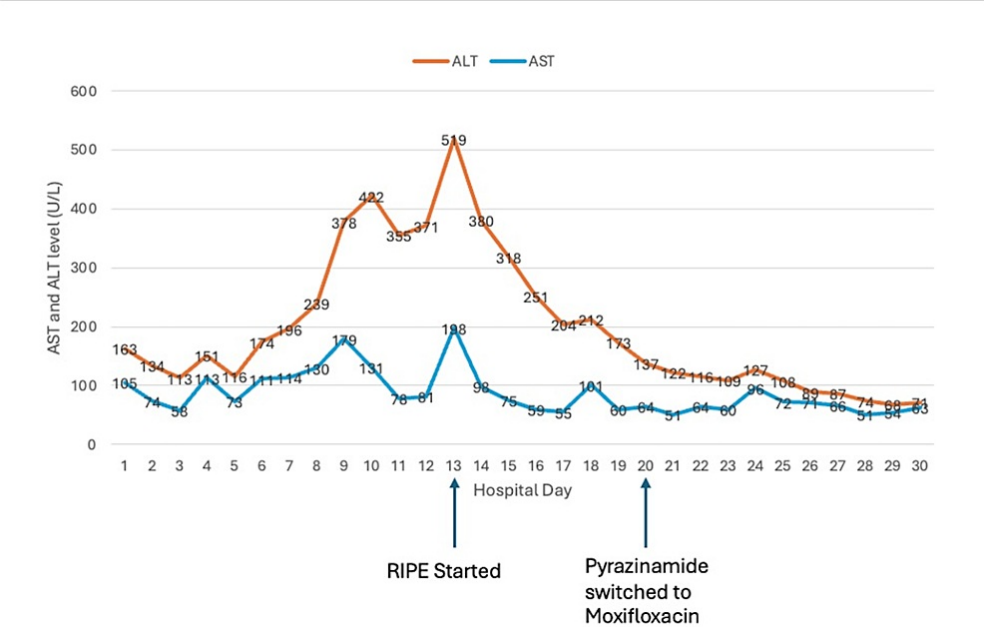
Trend of ALT and AST during the patient's hospitalization After starting RIPE on day 13 of hospitalization, both ALT and AST levels started to decline, suggesting hepatic involvement of tuberculosis. After an increase in ALT and AST levels on day 18 of hospitalization, pyrazinamide was switched to moxifloxacin on day 20 of hospitalization with continuous improvement of transaminitis. AST: aspartate aminotransferase; ALT: alanine aminotransferase; RIPE: rifampin, isoniazid, pyrazinamide, and ethambutol

Eventually, BAL and pleural fluid cultures grew *M. tuberculosis*. The serum TB polymerase chain reaction (PCR) became positive as well. A diagnosis of tuberculosis was made. Given his fever and persistent symptoms, the decision was made to initiate TB therapy. The patient was started on RIPE, and corticosteroids were discontinued. His AST and ALT remained elevated at 198 U/L and 519 U/L prior to therapy; however, his clinical status required therapy, and the decision was made to monitor LFT levels while initiating RIPE. The patient’s LFT levels started to trend down on RIPE therapy. However, he continued to remain febrile. He was started on prednisone due to concern for immune reconstitution inflammatory syndrome and the improvement of his fever. After five days on RIPE, his AST and ALT had increased to 64 U/L and 137 U/L, respectively. Therefore, pyrazinamide was switched to moxifloxacin due to concern for drug-induced hepatotoxicity (Figure 4).

The patient’s respiratory status and fever gradually improved on anti-tuberculous and corticosteroid therapy. He was eventually discharged on a steroid taper and anti-tuberculous therapy. Adalimumab was held given concern for immunosuppression in the setting of an active infection. The patient’s outpatient course was complicated by an episode of worsening LFT levels and hyperbilirubinemia that was thought to be a drug-induced liver injury. Therefore, isoniazid was discontinued, and the rifampin dose was decreased, which improved his LFT levels. The patient was instructed to continue rifampin, ethambutol, and moxifloxacin for a total of nine months. For AS, the patient continued to take low-dose prednisone with a plan to resume adalimumab. 

## Discussion

Hepatic TB is a rare manifestation that encompasses 1% of extrapulmonary cases of TB [1]. Primary hepatic TB is rare due to the low oxygen pressure of the liver parenchyma, creating an unfavorable environment for the mycobacterium [2]. Hepatic TB can present as isolated lesions or via hematogenous spread in cases of miliary TB [4]. The majority of patients with hepatic TB are often asymptomatic [2]. However, hepatic TB can also present with nonspecific symptoms such as fever, weight loss, night sweats, abdominal pain, hepatomegaly, diarrhea, and pyrexia. Patients can also present with transaminitis [4,5]. Hepatic TB is more common in immunocompromised patients. Tumor necrosis factor-⍺ inhibitors, especially adalimumab, are associated with a three- to four-fold increased risk of TB infection [3, 6]. As a result, societies, such as the National Psoriasis Foundation, call for yearly screening for TB in patients on TNF-⍺ therapy [3].

The diagnosis of hepatic TB includes a combination of imaging modalities, laboratory findings, and histopathologic findings. Computed tomography is often the preferred imaging modality [1]. Computed tomography findings could be multiple hypodense micronodules or hepatomegaly without any nodular lesions [4,7]. A biopsy can be considered in diagnosing hepatic TB. Cultures from sampled tissue can take up to six weeks to yield growth, and their sensitivity could be less than 10%. Histopathologic evidence of caseating granulomas on microscopy has a sensitivity of around 65% [4]. A PCR of the sampled tissue for *M. tuberculosis* DNA has a sensitivity of 53%-88% and a specificity of 96%-100% [4]. The preferred treatment regimen for hepatic TB remains to be RIPE, despite the potential hepatotoxicity of isoniazid, rifampin, and ethambutol. There is no consensus on the duration of treatment for hepatic TB. However, many studies have shown favorable outcomes with extended treatment up to 12 months [1].

## Conclusions

In our patient, the more common causes of hepatitis, such as viral, drug-induced, and autoimmune hepatitis, were ruled out. In addition, the results of the serum TB-PCR, pleural biopsy, and positive BAL and pleural fluid cultures for *M. tuberculosis* raised our suspicion for hepatic TB. Furthermore, the improvement of LFTs upon initiating an anti-tuberculous regimen supported the diagnosis of hepatic TB. Our case highlights the importance of considering hepatic TB in the differential diagnosis of patients on TNF-⍺ inhibitors who present with asymptomatic transaminitis. Tumor necrosis factor-⍺ inhibitors are often prescribed for the treatment of inflammatory bowel disease. Therefore, screening for TB prior to initiating TNF-⍺ inhibitors and annual surveillance for TB are vital in preventing serious infections in this group of patients.
